# Anesthetic management of the removal of a giant metastatic cardiac liposarcoma occupying right ventricle and pulmonary artery

**DOI:** 10.1186/1749-8090-9-56

**Published:** 2014-03-22

**Authors:** Jianhong Xu, Yueying Zheng, Liqing Wang, Qiang Feng, Ceyan Yu, Shengmei Zhu

**Affiliations:** 1 Department of Anesthesiology, The First Affiliated Hospital, School of Medicine, Zhejiang University, 79 Qingchun Road, Hangzhou 310003, China; 2 Department of Thoracicardiology, The First Affiliated Hospital, School of Medicine, Zhejiang University, 79 Qingchun Road, Hangzhou 310003, China

**Keywords:** Liposarcoma, Cardiac tumor, Hypotension, Cardiopulmonary bypass, Transesophageal echocardiography

## Abstract

A 60 years old chinese male scheduled for a removal of an intracardiac mass occupying majority of right ventricular space, right ventricular outflow tract and pulmonary artery. The giant cardiac mass was later diagnosed pathologically as metastatic liposarcoma. The patient had a history of surgical removal of myxoid liposarcoma from his left thigh many years ago. It is extremely rare for liposarcoma to metastatize to right ventricle and pulmonary artery. The anesthetic management of the surgical procedure to remove this kind of intracardiac mass poses significant challenges to anesthesia providers. Our patient developed refractory hypotension after induction of general anesthesia which necessitated urgent cardiopulmonary bypass. The surgical procedure was successful and the patient recovered from the surgery and was discharged home without significant complication. Accurate preoperative diagnosis and assessment of patient’s functional status, appropriate preoperative volume status, emergency cardiopulmonary bypass readiness, smooth and gentle induction of general anesthesia with less myocardial depressing agent, and closely monitoring patient’s vitals and hemodynamic parameters are imperative in managing this kind of patients.

## Background

Metastatic tumors to cardiac chamber(s) are uncommon. If it occurs, mostly it is due to renal cancer and metastasized to right atrium. Liposarcoma from lower extremity metastasized into right ventricle (RV) and protruded into pulmonary artery (PA) is extremely rare [1-4]. We report this case with the metastatic neoplasm occupying most RV chamber space and protruding into pulmonary artery. When we induced general anesthesia, the patient collapsed hemodynamically. Emergency cardiopulmonary bypass (CPB) was established. The patient underwent a successful removal of the metastatic tumor and recovered smoothly postoperatively. The complete resection of tumor is a recognized therapy with documented favorable prognosis [5]. The scheduled procedure in this kind of patient poses unique challenges to the anesthesiologist(s). We will discuss what we have learned from this case and the potential challenges anesthesia provider(s) will face.

## Case presentation

**Figure 1:**
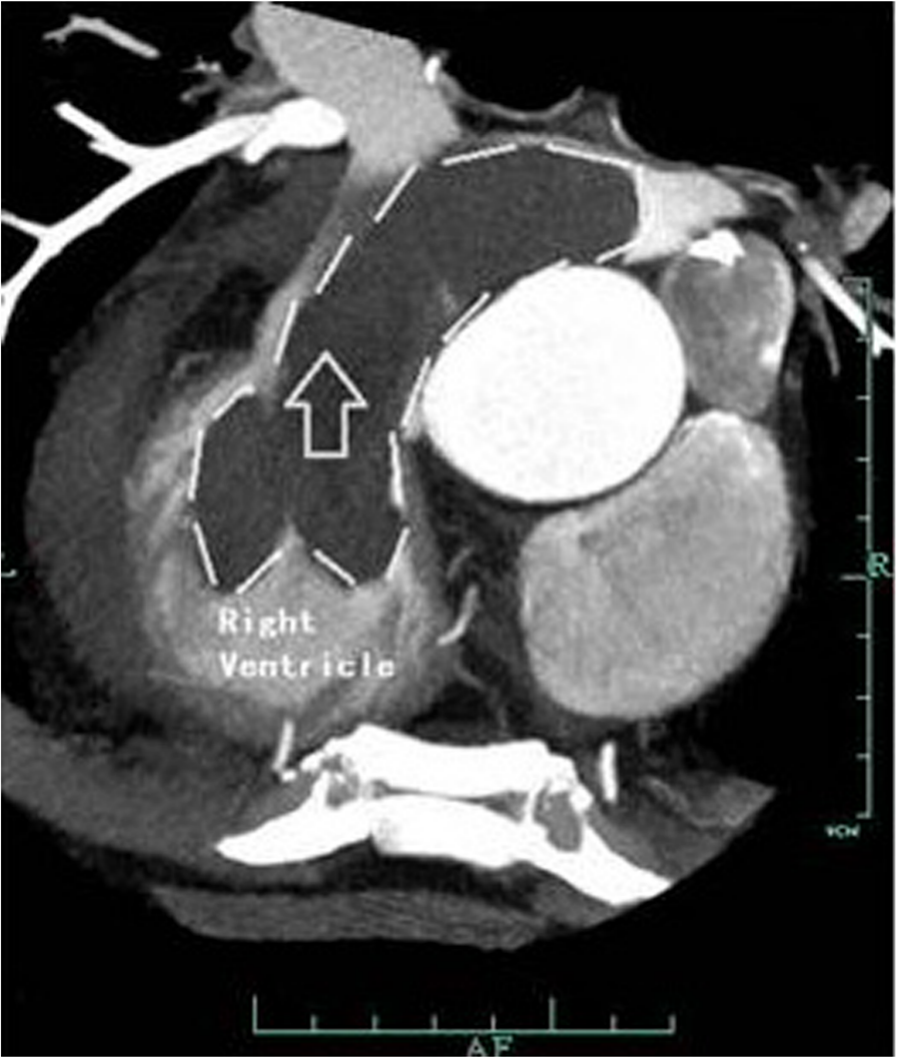
**Iodine enhanced computerized tomography of the patient’s chest.** Sagittal view confirmed a large filling defect in the right ventricle extended into the right pulmonary artery (arrow), rending almost complete occlusion of the right ventricular outflow tract.

A 60 years old chinese male was admitted to our hospital with chest tightness, dyspnea, systemic edema, and abdominal distension for more than 1 week. Physical examination is within normal limits except mild systematic edema. Electrocardiography (ECG) showed complete right bundle branch block and RV hypertrophy. The chest X-ray revealed cardiomegaly (cardiothoracic ratio: 60%). The transthoracic echocardiography detected a large echogenic mass (10.4*4.1 cm) which occupied most chamber space of RV and RV outflow tract (RVOT), was attached to the RV free wall, and extended into the main pulmonary artery and its bifurcations. A small pericardial effusion and left ventricular diastolic dysfunction were also detected. Computerized tomography (CT) with contrast of the chest confirmed the presence of the mass, which caused obvious obstruction of the RVOT (Figure 1). Laboratory results were unremarkable. The patient had a right bundle branch block for about 10 years and a surgical removal of a left thigh liposarcoma 20 years ago. With the preoperative diagnosis of intracardia tumor, the patient was scheduled for excision of the mass with CPB. On arrival to operating theatre, O_2_ (5 L/min) via face mask was administered and all American Society of Anesthesiologists (ASA) standard monitors were started. Pulse oxygen saturation (SpO_2_) was 98%, and heart rate 100 beats/min. The left radial arterial and large-bore intravenous access was obtained. The invasive blood pressure was 120/85 mmHg (mean blood pressure, MBP, 100 mmHg). A double-lumen central venous catheter (14, 18gauge, 13 cm length from the catheter tip to the skin; Arrow International, USA) was inserted to the right internal jugular vein before the induction of general anesthesia. Then the central venous pressure (CVP) was measured as 31 mmHg. Lactate Ringer’s solution 1000 ml was administrated, and then the induction of general anesthesia was performed slowly with midazolam 2 mg *iv*, etomidate 8 mg *iv*, rocuronium 50 mg *iv*, sufentanyl 30 μg *iv*, and scopolamine 0.3 mg *iv*. During induction, blood pressure fell about 40 mmHg from baseline, which responded to phenylephrine (40 μg, *iv*) and fluid administration. The results from arterial blood gas revealed pH 7.30, PaCO_2_ 37, PaO_2_ 89 (FiO_2_ at 50%). General anesthesia was maintained with propofol (200 mg/h) and cis-atracurium (10 mg/h), and sufentanyl (*iv,* intermittently according to hemodynamic changes). Meanwhile, dopamine (3-8 μg/kg/min) was given continuously. MBP decreased below 60 mmHg after sternal retraction, unresponsive to intravenous fluid and phenylephrine administration. Heart rates fluctuated around 90 beats/min. SpO_2_ maintained at 95-100%. CVP increased to 35 mmHg. The medication for maintenance of general anesthesia was turned down temporarily. About 24 minutes after induction of general anesthesia, urgent CPB was initiated after intravenous heparine was given. Ulinastatin (30,000 U) and methylprednisolone (40 mg) were also administrated. Then the surgery team removed the neoplasm successfully from the RV, RVOT and pulmonary artery. Total CPB and aortic cross-clamp times were 90 and 24 minutes, respectively. The patient was weaned from CPB easily with pace-maker and epinephrine infusion of 6 μg/min. At the termination of CPB, the pH was 7.30, PaCO_2_ 44 mmHg, PaO_2_ 276 mmHg with FiO_2_ 80%. After CPB, the patient’s heart rate was maintained at around 90 beats/min, blood pressure 90-100/50-60 mmHg, and CVP 9-11 mmHg. The patient was extubated at postoperative day one in surgical ICU (intensive care unit) and he was discharged home in a good condition 11 days postoperatively. The final histopathology report of the tumor mass revealed myxoid liposarcoma. The post-operative ECG indicated no residual mass was inside the heart chambers.

## Discussion

The occurrence of metastatic liposarcoma to the heart is rare. Metastatic liposarcoma occurs most likely in the lungs, liver, lymph node, brain and skeletal system [6]. Only 20 cases of metastatic cardiac liposarcoma were reported in the literature prior to our report [4,7-9]. Diagnosis of the isolated heart tumor as being metastatic is unusual unless the primary site is discovered. Liposarcoma is one of the most common soft tissue sarcomas, usually found in the lower extremities. The myxoid of histological classification accounts about 50% in liopsarcoma subgroups, which is characterized by a slow growth and a low risk for the metastasis. Some authors believe that it may take 3-25 years to present at a metastatic location [6]. In our patient, the interval between the appearance of primary tumor and metastasis was about 20 years.

Clinically, the symptoms of cardiac metastasis are basically nonspecific. Pericardium is usually involved and pericardial effusion often exists. Heart failure is a common clinical feature. And cardioembolic stroke, myocardial infarction and even death from malignant arrhythmia may be its serious complications. Generally, cardiac symptoms appear slowly in onset. Nevertheless, in our case, symptoms on admission were 1-week history of clinical symptoms related to the right heart failure. Two-dimensional ECG and CT of the chest are reportedly very accurate in making the diagnosis and assessing the extent of a cardiac mass preoperatively, though angiocardiography would better provide spatial and temporal resolution in this kind of patients [10]. The prognosis of the metastatic liposarcoma is usually poor. Surgery is indicated when complete resection of tumor is feasible and/or relieving intracardiac obstruction is necessary [11].

The concerns in anesthetizing these patients with tumors involved in the RV, the RVOT, and even PA include acute RV failure, hypoxemia, low cardiac output, tamponade, and potential pulmonary emboli. There is no “standard method” for the anesthetic management of this kind of surgical procedures. We believe the following strategies will help manage these patients:

1. Preoperative evaluation: accurate preoperative diagnosis, assessment of tumor size, location, involvement of cardiac valves, tumor mobility, degree of obstruction to blood flow etc and evaluation of patient’s overall cardiac functional status and comorbidities;

2. Preoperative volume replacement is very important, hypovolemia should be avoided;

3. Monitoring: non-thermodilution technique for cardiac output measurement should be used, because we cannot insert a pulmonary artery catheter for these patients. CVP is possible, but the catheter cannot be too close to superior vena cava-right atrium junction; Transesophageal ECG will be very helpful in multiple aspects: confirming diagnosis, assessment of the mass, evaluation of blood volume status, cardiac function and comorbidities, monitoring the evolving process of the surgical procedure. In addition, it is useful in detecting the tumor fragmentation, dislodgement or embolism [12,13]. Unfortunately we did not use TEE (transesophageal echocardiography) due to technical difficulties.

4. Emergency CPB readiness: sedating patient for preoperative invasive line placement, or inducing general anesthesia can all cause hemodynamic collapse, so emergency CPB readiness is mandatory. Surgical sterilizing and draping before the initiation of induction of anesthesia is also mandatory. In some report, some authors even suggested to prepare for femoral-femoral cannulation and bypass [12].

5. Smooth and slow-titration induction of general anesthesia with less myocardial depressing agent, and closely monitoring patient’s vitals and hemodynamic parameters are imperative in managing this kind of patients. Avoidance of sedating these patients outside of operation room (OR) is also very important because if patient’s hemodynamic parameters worsen, emergency CPB is necessary, it is more favorable if patient is inside the OR.

6. Judicious use of inotropes: inotropes are double-sides sword for these kinds of patients. When using appropriately, inotropes can enhance myocardial contractility and improve hemodynamics. On the other hand, inotropes can also cause increased contractility which leads to narrower passage for intracardiac blood flow.

We supplemented the patient with 1000 ml of crystalloid before the initiation of induction. However, we were not sure whether the RV preload was full, since the CVP monitoring might not accuratly reflect the RV volume status due to the RV mass. Despite of the slow and careful induction of anesthesia and intravenous infusion of dopamine for improving RV contractility, hypotension happened after induction and exacerbated after sternotomy. This hypotension might be related to decreased systemic vascular resistance (SVR), which caused decreased venous return, thus lower cardiac output and hypotension. Another potential mechanism is the sudden dilatation of RV. Flexman AM *et al* reported with TEE monitoring that the distended RV in the condition of acute RV failure forced the interventricular septum to be shifted the left, which reduced the left ventricle volume further [12]. In our case, the surgeon did find the heart congestion during the exploration. Undoubtedly, TEE measurements would be significantly useful and helpful for cardiac assessment and monitoring. In addition, delicate performance during surgery is also crucial for the patient. The surgery team adopted a special technique of suctioning tumor instead of excision avoid tumor fragmentation, thus reducing the chance of residual tumor or tumor migration to other tissue or organs. Post-operative ECG assesses the completeness of surgical removal and may reveal some unexpected findings.

## Conclusions

Giant metastatic cardiac liposarcoma occupying right ventricle and pulmonary artery is extremely rare. The anesthetic management of the surgical procedure to remove this kind of tumor poses significant challenges to anesthesia providers. Accurate preoperative diagnosis and assessment of patient’s functional status, appropriate preoperative volume status, emergency CPB readiness, smooth and gentle induction of general anesthesia with less myocardial depressing agent, and closely monitoring patient’s vitals and hemodynamic parameters are imperative in managing this kind of patients.

## Consent statement

Written informed consent was obtained from the patient for publication of this case report and any accompanying images. A copy of the written consent is available for review by the Editor-in-Chief of this journal.

## Abbreviations

ASA: American society of anesthesiologists; CPB: Cardiopulmonary bypass; CT: Computerized tomography; CVP: Central venous pressure; ECG: Electrocardiography; ICU: Intensive care unit; MBP: Mean blood pressure; OR: Operation room; PA: Pulmonary artery; RV: Right ventricle; RVOT: Right ventricular outflow tract; SpO2: Pulse oxygen saturation; SVR: Systemic vascular resistance; TEE: Transesophageal echocardiography.

## Competing interests

The authors declare that they have no competing interests.

## Authors’ contributions

XJH and ZSM carried out the anesthetic management of the patient. ZYY participated in the following-up of this patient and drafted the manuscript. WLQ participated in modify the manuscript and submitted to the journal. FQ and YCY participated in the operation of this patient. All authors read and approved the final manuscript.
